# Amprenavir Mitigates Pepsin-Induced Transcriptomic Changes in Normal and Precancerous Esophageal Cells

**DOI:** 10.3390/ijms26136182

**Published:** 2025-06-26

**Authors:** Pelin Ergun, Tina L. Samuels, Angela J. Mathison, Tianxiang Liu, Victor X. Jin, Nikki Johnston

**Affiliations:** 1 Department of Otolaryngology and Communication Sciences, Medical College of Wisconsin, Milwaukee, WI 53226, USA; pergun@mcw.edu (P.E.); tsamuels@mcw.edu (T.L.S.); 2 Mellowes Center for Genomic Science and Precision Medicine, Medical College of Wisconsin, Milwaukee, WI 53226, USA; amathison@mcw.edu; 3 Division of Biostatistics, Data Science Institute, MCW Cancer Center and Mellowes Center for Genome Science and Precision Medicine, Medical College of Wisconsin, Milwaukee, WI 53226, USA; tiliu@mcw.edu (T.L.); vjin@mcw.edu (V.X.J.); 4 Department of Microbiology and Immunology, Medical College of Wisconsin, Milwaukee, WI 53226, USA

**Keywords:** pepsin, gastroesophageal reflux, Barrett’s esophagus, esophageal adenocarcinoma, transcriptomic analysis

## Abstract

Gastroesophageal reflux disease (GERD) is associated with inflammatory and neoplastic changes in the esophageal epithelium. Despite widespread PPI use, esophageal adenocarcinoma (EAC) incidence continues to rise, implicating non-acidic reflux components such as pepsin in disease progression. We performed transcriptomic profiling to assess pepsin-induced changes and the protective effect of amprenavir in vitro. Het-1A (normal) and BAR-T (Barrett’s) cells (*n* = 3) were treated at pH 7.0 with pepsin and/or 10 μM amprenavir for 1 h. RNA-seq identified DEGs (FDR ≤ 0.05, |log₂FC| ≥ 0.375), and Ingenuity Pathway Analysis revealed enriched pathways. Pepsin exposure altered mitochondrial function, oxidative phosphorylation, epithelial integrity, signaling, and inflammatory pathways in both cell lines. Amprenavir attenuated these transcriptomic perturbations, preserving mitochondrial and stress-response pathways. Notably, BAR-T cells exhibited heightened activation of wound-healing and epithelial repair pathways, whereas Het-1A cells showed greater mitochondrial and systemic stress pathway alterations. Pepsin drives transcriptomic dysregulation in esophageal epithelial cells under non-acidic conditions, and amprenavir shows potential to counteract peptic injury. Further studies are needed to validate these findings and explore amprenavir’s therapeutic utility in GERD management and EAC prevention.

## 1. Introduction

Gastroesophageal reflux disease (GERD) is a chronic condition in which stomach contents, such as acid, pepsin, and bile acids, backflow to the esophagus, leading to irritation and inflammation [1]. It is a continuing public health concern typically linked to symptoms such as heartburn and regurgitation [2]. GERD is prevalent, impacting approximately 14% of the worldwide population, although prevalence varies considerably among various regions and nations [3,4]. It is also common in infants and young children [5]. In some GERD patients, extended contact with gastric substances causes metaplastic alterations in the esophageal lining, leading to Barrett’s esophagus (BE), a precancerous condition marked by columnar metaplasia of the esophageal epithelium containing goblet cells. Patients with GERD are three times more likely to develop esophageal adenocarcinoma (EAC), and this risk increases 30-fold in those with BE [6].

GERD is typically treated with proton pump inhibitors (PPIs), aimed at suppressing gastric acid secretion. Despite their popular use, there are concerns regarding their long-term safety such as bone fractures, gastric cancer, and chronic kidney disease risk [7]. Additionally, approximately 40% GERD patients have symptoms that are unresponsive to PPI treatment [8,9]. While PPIs are effective at suppressing stomach acid, they neither prevent gastric reflux nor protect against bile acids or pepsin, so patients continue to experience weakly acidic or non-acidic reflux and the resulting damage [8,10,11]. Importantly, exposure to refluxate regardless of acid content can trigger inflammatory responses, with interleukins such as IL-6, IL-8, and IL-1β, playing key roles as mediators of mucosal inflammation, contributing to tissue injury and the progression of GERD to BE and EAC [6,12,13,14,15,16]. Consequently, there is a growing tendency among GERD patients to seek alternative treatments to long-term PPI therapy [8,9,17]. Although anti-reflux surgery can provide effective symptom relief, it is primarily suitable for patients with pathological GERD, particularly those with severe erosive esophagitis [9,13,18].

**Figure 1:**
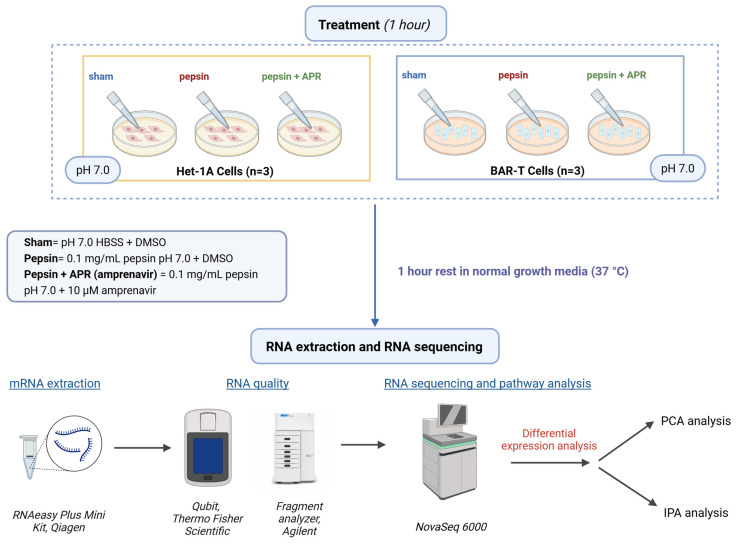
Experimental schema.

In light of the therapeutic challenges associated with PPIs, fosamprenavir, the prodrug of the FDA-approved HIV protease inhibitor amprenavir, has emerged as a potential novel treatment for pepsin-mediated mucosal damage [19,20]. Unlike bile acids, which are not consistently present in all refluxate, pepsin is a ubiquitous component of gastric reflux [11,14,21]. Therefore, targeting pepsin in addition to gastric acid may offer a more comprehensive approach, as pepsin can still exert harmful effects even in non-acidic environments [14,22,23]. Our group has recently discovered that the HIV protease inhibitor amprenavir could serve as a promising therapeutic agent targeting pepsin in laryngopharyngeal reflux (LPR). Amprenavir inhibits pepsin activity at low micromolar concentrations, and its prodrug, fosamprenavir, successfully prevented histological alterations in an LPR mouse model [19]. Concurrently, amprenavir has been shown to protect against epithelial barrier disruption, E-cadherin cleavage, and matrix metalloproteinase dysregulation in both esophageal and laryngeal cells exposed to pepsin at pH 4 [24,25]. To better understand the protective role of amprenavir against peptic injury in PPI-recalcitrant GERD, we investigated whether amprenavir could prevent transcriptomic alterations caused by pepsin in normal human esophageal cells and precancerous BE cells. Amprenavir’s protective role against pepsin and chemopreventive potential in esophageal cell cultures could provide a dual benefit: a more comprehensive GERD management strategy and a reduction in the progression to EAC. In this study, we conducted RNA-seq profiling in human normal esophageal epithelial cells (Het-1A) and Barrett’s esophagus cells (BAR-T), which are well-established models for GERD and BE, respectively, as previously published [24,26,27,28,29]. A one-hour refluxate exposure was employed, consistent with prior studies in both normal and cancerous esophageal cells [30,31] (Figure 1). We sought to identify pepsin-mediated genome-wide transcriptomic changes in normal esophageal and BE cells in vitro and to investigate the protective potential of amprenavir as a treatment option for PPI-refractory GERD patients.

## 2. Results

**Figure 2:**
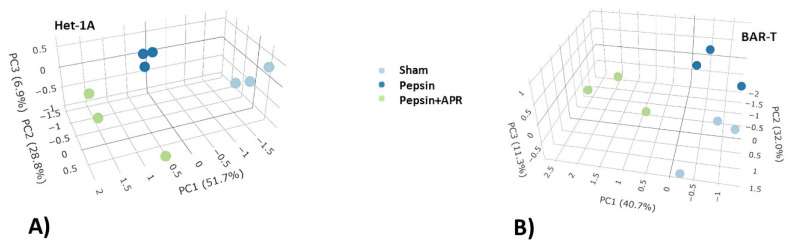
PCA of RNA-seq data from Het-1A and BAR-T cells under different treatment conditions. (**A**) PCA of Het-1A cells treated with pepsin (dark blue), pepsin + amprenavir (green), and sham (pH 7.0 HBSS; light blue). (**B**) PCA of BAR-T cells treated with pepsin (dark blue), pepsin + amprenavir (green), and sham (light blue). PCA = principal component analysis; APR = amprenavir.

RNA-seq profiling resulted in an average of 119,963,203 pair-end reads per Het-1A sample, with 51,412,518 reads mapping to exons/genes, while there was an average of 105,615,779 reads per BAR-T sample, with 47,188,950 reads mapping to exons/genes. Quality control confirmed high-quality sequencing data, and DE analysis was conducted using a data-driven approach. In Het-1A cells, principal component 1 (PC1) explained 51.7% of the total gene expression variance, with PC2 accounting for 28.8%, and PC3 for 6.9% (Figure 2A). In the PCA plot, each sample is color-coded by treatment (light blue = sham, dark blue = pepsin, green = pepsin + APR) and labelled with its replicate number (1–3), making it clear which points belong to each group. Similarly, in BAR-T cells, PC1 explained 40.7% of the variance, PC2 accounted for 32.0%, and PC3 for 11.3% (Figure 2B), with the same color- and replicate-label scheme. The number of significantly DEGs among treatment conditions is shown in Supplementary Figures S1 and S2. The tables of pairwise DE analysis are provided in Supplementary Tables S1 and S2 for both cell lines. Heatmaps illustrating the conditions and replicates for pairwise comparisons are provided in Supplementary Figures S3 and S4. Complete names of all gene symbols below are provided in Appendix A.

In Het-1A cells, the IPA analysis of DEGs revealed that the top canonical pathways significantly affected included Class A/1 (Rhodopsin-like receptors), Natural Killer Cell Signaling, and Cell Surface Interactions at the Vascular Wall, along with key molecular and cellular functions impacted by pepsin (Table 1). Metabolic Disease was a common top disease/disorder across all comparisons. Cellular Development, Cellular Growth and Proliferation, and Cell Death and Survival were the top molecular and cellular functions in the pepsin vs. sham and pepsin + amprenavir vs. sham comparisons. In the pepsin + amprenavir vs. sham comparison, the top canonical pathways included Hematoma Resolution Signaling, rRNA Processing, and NGF-stimulated Transcription, while Oxidative Phosphorylation, Mitochondrial Dysfunction, and rRNA Processing were prominent in the pepsin alone comparison. The top upstream regulators were nelfinavir in pepsin vs. sham, *HGF* in pepsin + amprenavir vs. sham, and *DAP3* in the pepsin + amprenavir vs. pepsin comparison. The top analysis-ready molecules from the comparisons are presented in Table 2.

In BAR-T cells, the IPA analysis of DEGs showed that the Wound Healing Signaling Pathway was a top canonical pathway across all comparisons. In the comparison of pepsin + amprenavir vs. pepsin treatment, HMGB1 Signaling and Macrophage Classical Activation Signaling Pathways were also significantly activated (Table 3). Additionally, Keratinization and Glucocorticoid Receptor Signaling pathways, along with molecular and cellular functions related to Cell Morphology, Cellular Assembly and Organization, and Cellular Function and Maintenance, were prominently triggered in both the pepsin vs. sham and pepsin + amprenavir vs. sham comparisons. Cancer pathways were a common feature in the sham and pepsin treatments compared to the pepsin + amprenavir group. The top upstream regulators were *CAMK4* in pepsin vs. sham, *GSTP1* in pepsin + amprenavir vs. sham, and TBK1 in the pepsin + amprenavir vs. pepsin comparison. Table 4 displays the top analysis-ready molecules from the comparisons.

## 3. Discussion

Designed to inhibit gastric acid production, PPIs remain central to treatment but are associated with adverse effects like calcium malabsorption and increased osteoporosis risk [32,33,34]. Long-term PPI therapy has also been linked to impaired iron and vitamin B₁₂ absorption due to gastric alkalinization [7], as well as intestinal dysbiosis—factors that may contribute to colorectal neoplasia [35]. Although PPIs are the primary therapy for GERD, over 40% of patients continue to experience symptoms [36,37]. Moreover, newer and more potent acid-suppressing medications, such as potassium-competitive acid blockers, have not shown superior efficacy [38,39]. This indicates GERD involves mechanisms beyond acid alone. Despite their widespread use, PPIs have not reduced reflux-related cancers, including EAC, highlighting their limited impact on carcinogenesis [40,41]. They also fail to lower EAC risk in BE patients [42].

BE is marked by the replacement of squamous epithelium with columnar epithelium containing goblet cells, a response to chronic reflux injury and irregular healing [43,44]. In addition to acid, the gastric protease pepsin is consistently present in refluxate and plays a major role in esophageal damage [11,45,46]. The esophagus lacks a protective mucus layer against pepsin, which degrades extracellular matrix and epithelial membranes, causing inflammation and tissue injury [12,14,21,22,45]. Many inflammatory and neoplastic conditions of the aerodigestive tract have been associated with pepsin [15,21,47,48], and substantial evidence suggests it can induce inflammation and injury to the epithelium even in the absence of acid [22,23,26,47,49,50]. Work by our group and others has shown that pepsin can be detrimental even at a neutral pH [14,22,23,26,51,52,53,54], and the pathophysiological contribution of non-acidic pepsin to the progression of BE and EAC has been revealed [15,30,49].

These findings emphasize the need for GERD treatments that target pepsin. We recently identified amprenavir as an effective pepsin inhibitor through a high-throughput small-molecule screening platform, protein crystallography, and biochemical activity assays [19]. Although originally approved by the FDA as an HIV-1 protease inhibitor, our in vitro and in vivo experiments have supported the capacity of amprenavir to inhibit pepsin and protect the aerodigestive tract from its deleterious effect [19,24,25]. Through transcriptomic analysis, this study aimed to explore gene interactions, regulators, molecular functions, and key pathways influenced by pepsin in normal esophageal and BE cells in vitro and the potential of amprenavir as a protective treatment for GERD patients unresponsive to PPIs.

### 3.1. Pepsin Causes Cell Injury and Disrupts Cytoskeletal Organization

It was initially believed that pepsin would only cause mucosal damage in acidic conditions [45]; however, it is now known that pepsin causes damage in non-acidic reflux conditions. The enzyme remains stable at neutral pH and can be reactivated within acidic intracellular vesicles in epithelial cells, in which pepsin can be retained for more than a day [22]. In this study, we observed significant transcriptomic alterations in both Het-1A and BAR-T cells exposed to pepsin, highlighting the diverse biological impacts of pepsin at pH 7.0 in GERD and Barrett’s esophagus mimicking on-PPI conditions.

In Het-1A cells, the top canonical pathways such as “Class A/1 (Rhodopsin-like receptors)”, “Natural Killer Cell Signaling”, and “Cell Surface Interactions at the Vascular Wall” suggest that pepsin disrupts epithelial signaling, immune modulation, and barrier integrity, contributing to early dysfunction (Table 1) [55,56,57]. These pathways are critical in maintaining the integrity of epithelial barriers, immune response, and cellular communication. The “Class A/1 (Rhodopsin-like receptors)” pathway is involved in signaling mechanisms that regulate cell migration, survival, and differentiation, processes crucial in response to epithelial injury and inflammation [58]. “Natural Killer Cell Signaling” is vital for immune surveillance and cytotoxic activity, and disruption in this pathway may lead to impaired immune responses in the context of chronic inflammation, as seen in impaired esophageal tissues [59,60,61]. “Cell Surface Interactions at the Vascular Wall” plays a role in endothelial function, where disruption can contribute to vascular changes associated with inflammatory diseases like GERD [62]. These changes are associated with metabolic, gastrointestinal, and organismal abnormalities, emphasizing pepsin’s impact on cellular development and growth. The gene expression changes following pepsin exposure reveal a profound disruption of key cellular pathways related to stress responses, tissue integrity, and immune activation. In accordance with the observed disruption in the “Cell Surface Interactions at the Vascular Wall” and “Natural Killer Cell Signaling” pathways, the downregulation of genes such as *CHAC1*, *SESN2* and *SLC7A11* points to a decrease in oxidative stress regulation, cellular survival, and extracellular matrix remodeling (Table 2) [63,64,65]. The idea that pepsin disrupts normal epithelium function and causes early epithelial dysfunction in GERD is supported by these data. Furthermore, in line with the previously noted “Immune Response” pathways, the increase in *HSPA1A/HSPA1B* and *CXCL14* suggests an active stress response and inflammatory signaling [66,67,68,69]. According to these changes, exposure to pepsin may cause tissue damage and a series of immunological reactions, which could exacerbate the inflammatory nature of GERD and lead to pathological modifications in esophageal epithelial cells [14,19,24,26,30]. Nelfinavir, another aspartic protease inhibitor used for the treatment of HIV/AIDS, was identified as a top upstream regulator in the pepsin vs. sham comparison (Table 1), supporting the capacity of peptidomimetics in this drug category to modulate pepsin-induced cellular damage [20].

Downregulation of genes, including *FOSB* and *KRT6B*, affected transcriptional regulation and keratinization in BAR-T cells, causing clear alterations indicative of metaplastic transformation (Table 4) [70,71]. These alterations are consistent with the deregulation of the “Wound Healing Signaling” and “Keratinization” pathways, emphasizing the disruption of epithelial differentiation and repair processes that are typical of BE (Table 3) [43,72]. Wound Healing Signaling is a crucial pathway involved in tissue repair and inflammation; its activation in response to pepsin exposure suggests a maladaptive healing response in GERD and BE [43]. Keratinization pathways are critical for epithelial cell differentiation and integrity; dysregulation here points to changes characteristic of metaplasia, a key feature in BE progression [73,74]. These findings are in alignment with our previous studies in human esophageal and hypopharyngeal carcinoma cells, which demonstrated that non-acidic pepsin enhances scratch wound-healing (a product of cell migration and proliferation) and promotes changes in esophageal cell cytokeratin expression profile that are consistent with transition from normal to BE phenotype [15,30]. Given that increased cell migration and proliferation are hallmarks of wound healing, chronic inflammation, and tumor metastasis, these findings reinforce the notion that pepsin contributes to pathological wound healing responses. Additionally, the upregulation of keratin genes, which are involved in epithelial remodeling, further supports a shift toward a metaplastic phenotype in response to pepsin exposure [75,76]. A stress-adaptive survival mechanism is also suggested by the activation of “Glucocorticoid Receptor Signaling”, with upstream regulators such as *CAMK4* implicated in maintaining cell viability in a dysregulated environment [77,78,79]. Collectively, these findings suggest that pepsin not only impairs normal epithelial homeostasis but also promotes metaplastic reprogramming, potentially contributing to the pathogenesis of EAC [15,30].

### 3.2. Amprenavir Enhances Repair Pathways and Supports Epithelial Integrity Against Peptic Damage

The comparison between pepsin and amprenavir groups in Het-1A cells emphasizes amprenavir’s function in reducing damage caused by pepsin, especially via pathways including “rRNA Processing”, “Oxidative Phosphorylation”, and “Mitochondrial Dysfunction” (Table 1). Activation of these pathways indicates that amprenavir mitigates pepsin-induced alterations in cellular energetics and mitochondrial function, both of which are crucial in GERD and BE dysplasia [80,81,82]. The downregulation of mitochondrial genes *(MT-ND1*, *MT-ND2*, *MT-ND4*, *MT-CO1*, *MT-CO2*, *MT-CYB*) suggests a reduction in oxidative stress and electron transport chain dysfunction, processes linked to esophageal carcinogenesis (Table 2) [80,82,83,84,85]. This downregulation may reflect a compensatory response aimed at limiting pepsin-induced reactive oxygen species (ROS) production and preserving mitochondrial and epithelial integrity [68,86], similar to mechanisms observed in bile acid reflux models [87]. Future studies should directly measure reactive oxygen species production and mitochondrial membrane potential to validate amprenavir’s proposed reduction in oxidative stress in normal esophageal cells. Furthermore, reduced expression of *FOS*—a proto-oncogene involved in cellular stress responses—indicates damage [88]. The potential of amprenavir to reduce inflammation is highlighted by the decreased expression of acute-phase reactants *SAA2* and *SAA2-SAA4* [89]. The identification of upstream regulators such as *mtRNase P*, *DAP3*, and *NSUN3* may highlight the control of translational processes and mitochondrial ribosomal biogenesis [90,91,92], which are essential for reducing mitochondrial dysfunction brought on by pepsin exposure. Furthermore, amprenavir’s function in promoting regular cell functioning under neutral conditions is demonstrated by the comparison of pepsin + amprenavir against sham in those cells. Its role in supporting epithelial survival and repair is reflected in important pathways such as “Hematoma Resolution Signaling” and “NGF-stimulated Transcription” [93,94]. Increased protective mechanisms against cellular stress and apoptotic pathways suggest that amprenavir strengthens natural epithelium repair processes while reducing pepsin-mediated damage, supporting the hypothesis that it may help reduce the risk of progression from GERD to BE, which warrants further investigation in in vivo or clinical settings.

In BAR-T cells, involvement of amprenavir in wound healing and immune modulation is evident by the activation of the “Wound Healing Signaling Pathway”, “HMGB1 Signaling”, and “Macrophage Classical Activation Signaling Pathway” in the pepsin + amprenavir versus pepsin analysis (Table 3). HMGB1 Signaling is known to drive inflammatory responses and epithelial regeneration in reflux injury [95], while classical (M1) macrophage activation fosters clearance of damaged cells and matrix remodeling in GERD mucosa [96]. The downregulation of *RND1* and *REL*, which are involved in actin cytoskeleton remodeling and NF-κB-mediated inflammation, respectively [97,98], indicates amprenavir’s function in lowering inflammation and maintaining structural integrity (Table 4). Amprenavir-induced expression of *RGCC* (a regulator of endothelial and epithelial repair) suggests improved cell regulation [99].

Notably, although the Wound Healing Signaling pathway was also dysregulated in the pepsin versus control comparison, the specific genes driving this response differed. In the pepsin-treated group, keratin-related alterations were prominent—a pattern consistent with epithelial stress and early metaplastic remodeling [100], as indicated by IPA analysis. In contrast, the pepsin + amprenavir group exhibited broader modulation of inflammation-related genes, including downregulation of proinflammatory mediators such as *CXCL8, IL1A,* and *PDGFB*, and upregulation of genes involved in tissue remodeling and repair, including *CLCF1* [101] and *VIM* [102] (Supplementary Table S2). Both *CXCL8 (IL-8)* and *IL1A* are key cytokines involved in GERD-related inflammation. *IL1A* contributes to epithelial injury and immune cell recruitment during inflammation [103], while *CXCL8* promotes neutrophil activation and is consistently elevated in GERD and BE mucosa [12,104,105]. The suppression of these mediators by amprenavir indicates a shift from a proinflammatory to a more reparative immune environment. These distinct gene expression profiles suggest that amprenavir may redirect the wound healing response away from a proinflammatory, stress-associated state and toward a more regenerative and immunomodulatory repair process. These alterations correspond with GERD and BE pathology, where immune modulation and epithelial reconstruction are essential reactions to prolonged damage. Conditions linked to these pathways, such as gastrointestinal and dermatological diseases, emphasize the importance of these discoveries in relation to GERD and its progression to BE.

### 3.3. Summary and Limitations

In summary, our results underscore the disruptive impact of pepsin on epithelial cell activity, even in neutral pH environments, supporting prior reports of its ability to damage mitochondrial integrity, induce oxidative stress, and modify essential pathways linked to cellular signaling and tissue remodeling. In Het-1A cells, damage caused by pepsin was primarily associated with mitochondrial dysfunction and oxidative phosphorylation, along with dysregulation of genes involved in energy metabolism and cellular stress responses. In contrast, BAR-T cells exhibited alterations in pathways related to wound healing, immune regulation, and epithelial remodeling, emphasizing the distinct response profiles of BE-like cells to peptic injury. Amprenavir demonstrated protective effects in both cell types, reducing pepsin-induced epithelial damage and promoting tissue regeneration. Although this study is limited to transcriptomic profiling, results herein, such as pepsin-induced changes in mitochondrial dysfunction [15,46], oxidative stress [86], cell proliferation [106,107], and wound-healing [15,46] have been demonstrated in prior work. While it is difficult to estimate the concentration of pepsin that would be found in refluxate, the dose of pepsin used in this study is congruent with prior models of GERD and 10-fold less than that observed in the stomach of healthy subjects. Notably, the concentration of pepsin in the stomach can be elevated by 3-fold or more in patients taking PPIs; thus, our treatment dose may be considerably lower than that found in refluxate [108,109,110]. The concentration of amprenavir in this study is congruent with that found in the serum of patients taking the manufacturer’s recommended dose for treatment of HIV [19,111,112]. Our prior experiments support local conversion of fosamprenavir to active amprenavir [19], and we therefore expect to achieve even greater esophageal epithelial concentrations via a mucoadhesive formulation designed to prolong contact with the esophageal surface. One limitation of the study is that experiments were conducted using an in vitro model, which may not fully capture the complexity of in vivo tissue responses or replicate physiological conditions. We used only cell lines in this study; we acknowledge that our findings may be limited to these cell lines and may not fully represent the diversity of responses in primary cells or tissues, highlighting the need for future studies. Additional studies using in vivo models and clinical specimens will be helpful in confirming the findings and determining their relevance to patient care.

Furthermore, since this work is purely laboratory-based, we strongly encourage clinical follow-up studies involving patients often managed by gastroenterologists, with integrated nutritional interventions guided by dietitians or nutritionists. This approach is particularly relevant given that certain foods are known to exacerbate gastroesophageal reflux, and such nutritional care is already an established component of oncology teams in tertiary hospitals. This important clinical link bridges the essential laboratory research with everyday practice and patient management [113].

These data provide valuable insights into the molecular mechanisms underlying pepsin-induced epithelial injury and support the potential value of amprenavir in mitigating pepsin-driven epithelial damage in GERD and BE. Moreover, these data will guide our future research by prioritizing key signaling and metabolic pathways for functional analysis and therapeutic targeting in GERD and BE.

## 4. Materials and Methods

### 4.1. Cell Culture and Treatment

Immortalized human esophageal epithelial cells (Het-1A; American Type Culture Collection, Manassas, VA, USA) and hTERT-immortalized Barrett’s esophagus cells (BAR-T, kindly provided by Rhonda Souza [29]), were maintained in Bronchial Epithelial Cell Growth Medium (Sigma Aldrich, St. Louis, MO, USA) and Keratinocyte Growth Medium 2 (KGM; Lonza, Walkersville, MD, USA), respectively. BEGM was supplemented with 1x Antibiotic-Antimycotic (ThermoFisher Scientific, Waltham, MA, USA). KGM was supplemented with hydrocortisone, insulin, and transferrin, as provided, along with 180 μM adenine and 10 ng/mL cholera toxin (Sigma-Aldrich), 70 μg/mL bovine pituitary extract, 5% fetal bovine serum, and 1× Antibiotic-Antimycotic (ThermoFisher Scientific, Waltham, MA, USA). BAR-T were cultured on collagen-I coated plasticware (Biocoat; Corning, Corning, NY, USA), Het-1A on uncoated plasticware. Upon reaching 75% confluency, they were subjected to the designated pretreatment conditions in triplicate wells. Unless noted, cultures were treated in triplicate with Hanks’ Balanced Salt Solution (HBSS) at pH 7 to mimic on-PPI conditions, with or without 0.1 mg/mL porcine pepsin (Sigma Aldrich) and/or 10 μM amprenavir (Sigma Aldrich; in dimethyl sulfoxide [DMSO]) or equivalent volume of DMSO (solvent control) at 37°C and 5% CO_2_ for 1 h. Following treatment, the cells were washed twice with HBSS and incubated in normal growth media at 37 °C and 5% CO_2_ for an additional hour before harvest to extract the total RNA. Total RNA was extracted using the RNeasy Plus Mini Kit (Qiagen, Hilden, Germany), which eliminates genomic DNA, with QIAshredder columns. RNA quality was evaluated through UV spectroscopy (Nanodrop 2000; ThermoFisher Scientific), fluorimetry (Qubit; ThermoFisher Scientific), and a high-sensitivity RNA fragment analyzer (Agilent, Santa Clara, CA, USA) run (Figure 1).

### 4.2. RNA-seq and Ingenuity Pathway Analysis

RNA libraries were prepared using the Illumina TruSeq Stranded mRNA kit with dual indexing and sequenced on the Illumina NovaSeq 6000 platform, generating 100 bp paired-end reads at the Mellowes Center for Genomic Science and Precision Medicine (RRID:SCR_022926), as previously described [49]. Next, the RNA-seq data analysis was performed by the Bioinformatics Resource at the Mellowes Center as follows: FastQC [114] and RSeQC [115] were first used to perform quality control on the sequencing raw reads; across all 18 samples, read lengths ranged from 35 to 101 bp. On average, each sample yielded 112,789,491 total reads (≈56,394,745 read pairs). Of these, an average of 53,203,410 read pairs (≈94%) aligned successfully to the reference genome, including 36,291,879 junction-spanning pairs, indicating robust splice detection. Gene quantification resulted in an average of 49,283,871 gene counts per sample, and exon-level counts averaged 250,009,723, reflecting deep coverage of annotated features. Mean GC content was 72.1% for read 1 and 67.6% for read 2. Read duplication rates averaged 50.1% and 50.3%, respectively, indicating consistent library complexity. These metrics confirm the high quality and uniformity of the RNA-seq data across all conditions and replicates (Supplementary Table S3). 

*MAPRSeq3* workflow integrates a suite of open-source bioinformatics tools along with in-house developed methods to analyze paired-end RNA-Seq data [116]. Read alignment is performed with Star [117]. The BAM file is processed using featureCounts to summarize expression at gene and exon level. In addition to raw gene and exon expression counts, MAP-RSeq also provides normalized values (RPKM). This approach produced gene and exon counts after normalization by sequencing depth and gene length (linear reads per kilobase of transcript per million). Differential expression (DE) analysis was conducted using a pairwise approach with EdgeR [118] with the following thresholds: a minimum of one read per million in at least three samples, an adjusted *p*-value of ≤0.05, and an absolute fold change (FC) of ≥1.3 or [log2(FC) ≥ 0.3785]. Differentially expressed genes (DEGs) were then obtained and further analyzed with Ingenuity Pathway Analysis (IPA; Qiagen) and principal component analysis (PCA). In IPA, the statistical significance of enriched Canonical Pathways, upstream regulators, and Diseases or Functions is calculated using a right-tailed Fisher’s Exact Test, based on the overlap between the input gene list and gene sets curated in the IPA Knowledge Base [119]. The “overlap” refers to the proportion of DEGs in the dataset that intersect with a given pathway or regulator’s known targets. These *p*-values are reported as −log₁₀ (*p*-value) in IPA output, with a threshold of ≥1.3 (equivalent to *p* ≤ 0.05) considered statistically significant.

## 5. Conclusions

In conclusion, this study provides evidence that amprenavir protects against the pepsin-induced injury in a model that mimics PPI-treated GERD, in which pepsin is injurious at neutral pH. The data suggest that pepsin exposure will result in severe cell damage, including mitochondrial dysfunction, oxidative stress, and inflammatory responses, despite the use of PPIs. These findings lend further support to the hypothesis that refluxed pepsin, independent of acid, acts as an important contributor to epithelial injury and dysfunction in GERD. In this model of BE progression, pepsin exposure activates signaling pathways related to immune modulation, wound healing, and epithelial remodeling. Amprenavir, by inhibiting pepsin activity, mitigated these effects, promoting epithelial repair and integrity. These findings elucidate the role that pepsin continues to play in PPI-treated GERD and its likely contribution to the progression from normal to BE and EAC despite PPI therapy. The findings herein support the therapeutic value of pepsin-inhibiting amprenavir, and future studies are needed to better understand its rescue of epithelial integrity, energy metabolism, inflammatory response, and cell differentiation during PPI recalcitrant GERD.

## Figures and Tables

**Table 1:** Ingenuity Pathway Analysis of DEGs in Het-1A cells.

**Pepsin vs. Sham**		
**Top Canonical Pathways**	***p*-Value**	**Overlap**
Class A/1 (Rhodopsin-like receptors)	3.53 × 10^−3^	4/317 (1.3%)
Natural Killer Cell Signaling	7.06 × 10^−3^	3/198 (1.5%)
Cell surface interactions at the vascular wall	8.40 × 10^−3^	2/64 (3.1%)
**Top Diseases**	***p*-value range**	**#Genes**
Metabolic Disease	1.71 × 10^−2^–1.01 × 10^−6^	22
Organismal Injury and Abnormalities	1.71 × 10^−2^–1.01 × 10^−6^	48
Gastrointestinal Disease	1.71 × 10^−2^–2.60 × 10^−6^	45
**Molecular and Cellular Functions**	***p*-value range**	**#Genes**
Cellular Development	1.71 × 10^−2^–6.38 × 10^−6^	27
Cellular Growth and Proliferation	1.71 × 10^−2^–6.38 × 10^−6^	27
Cell Death and Survival	1.68 × 10^−2^–3.44 × 10^−5^	23
**Top Upstream Regulators**	***p*-value range**	
nelfinavir	9.27 × 10^−9^	
EIF2S1	6.11 × 10^−8^	
tosedostat	2.89 × 10^−7^	
**Pepsin + Amprenavir vs. Sham**		
**Top Canonical Pathways**	***p*-value**	**Overlap**
Hematoma Resolution Signaling Pathway	2.34 × 10^−8^	7/258 (2.7%)
rRNA processing	6.65× 10^−8^	4/32 (12.5%)
NGF-stimulated transcription	1.51 × 10^−7^	4/39 (10.3%)
**Top Diseases**	***p*-value range**	**#Genes**
Organismal Injury and Abnormalities	3.70 × 10^−3^–8.37 × 10^−9^	30
Cancer	3.70 × 10^−3^–2.66 × 10^−8^	30
Metabolic Disease	3.58 × 10^−3^–7.57× 10^−8^	21
**Molecular and Cellular Functions**	***p*-value range**	**#Genes**
Cellular Development	3.70 × 10^−3^–2.24 × 10^−9^	18
Cellular Growth and Proliferation	3.70 × 10^−3^–2.24 × 10^−9^	18
Cell Death and Survival	3.70 × 10^−3^–9.54 × 10^−8^	17
**Top Upstream Regulators**	***p*-value range**	
HGF	3.14 × 10^−15^	
GLI1	3.07 × 10^−13^	
PRL	1.86 × 10^−12^	
**Pepsin + Amprenavir vs. Pepsin**		
**Top Canonical Pathways**	***p*-value**	**Overlap**
rRNA processing	1.16 × 10^−24^	10/32 (31.2%)
Oxidative Phosphorylation	1.92 × 10^−16^	9/112 (8.0%)
Mitochondrial Dysfunction	5.26 × 10^−12^	9/344 (2.6%)
**Top Diseases**	***p*-value range**	**#Genes**
Developmental Disorder	1.46 × 10^−2^–8.74 × 10^−22^	14
Metabolic Disease	1.97 × 10^−2^–8.74 × 10^−22^	13
Neurological Disease	2.06 × 10^−2^–8.74 × 10^−22^	15
**Molecular and Cellular Functions**	***p*-value range**	**#Genes**
Cell-To-Cell Signaling and Interaction	1.97 × 10^−2^–8.81 × 10^−17^	10
Cell Signaling	1.33 × 10^−2^–1.70 × 10^−7^	6
Post-Translational Modification	1.33 × 10^−2^–1.70 × 10^−7^	7
**Top Upstream Regulators**	***p*-value range**	
DAP3	2.48 × 10^−30^	
NSUN3	3.80 × 10^−26^	
mtRNase P	1.13 × 10^−25^	

IPA enrichment of DEGs from pepsin vs. sham, pepsin + amprenavir vs. sham, and pepsin + amprenavir vs. pepsin, showing top pathways, diseases/functions, and upstream regulators with Fisher’s exact test *p* values and gene overlap (%).

**Table 2:** Top DEGs in Het-1A Cells.

Pepsin vs. Sham	logFC		Pepsin + Amprenavir vs. Sham	logFC		Pepsin + Amprenavir vs. Pepsin	logFC	
**CHAC1**	−1.74	↓	**CHAC1**	−1.37	↓	**SAA2**	−0.42	↓
**STC2**	−0.46	↓	**POTEE/POTEF**	−8.51	↓	**FOS**	−0.53	↓
**SESN2**	−0.57	↓	**COL1A2**	−6.29	↓	**MT−ND1**	−0.59	↓
**SLC7A11**	−0.60	↓	**FOS**	−0.57	↓	**SGPP2**	−0.39	↓
**COL1A2**	−3.99	↓	**CAV1**	−5.52	↓	**MT−CO2**	−0.47	↓
**POTEE/POTEF**	−4.25	↓	**HSPA1A−B**	0.71	↑	**MT−ND2**	−0.53	↓
**CAV1**	−4.42	↓	**HES1**	0.51	↑	**SAA2−SAA4**	−0.47	↓
**ADM2**	−0.68	↓	**MIDN**	0.47	↑	**MT−ND4**	−0.59	↓
**HSPA1A-B**	0.59	↑	**ID1**	0.54	↑	**MT−CYB**	−0.50	↓
**CXCL14**	0.41	↑	**IL11**	0.68	↑	**MT−CO1**	−0.41	↓

List of differentially expressed genes (DEGs) with log fold changes (logFC) in Het-1A cells under different conditions: pepsin vs. sham, pepsin + amprenavir vs. sham, and pepsin + amprenavir vs. pepsin. Genes with upregulation are marked with ↑, and downregulation with ↓. The table shows the logFC for each DEG under the respective conditions.

**Table 3:** Ingenuity Pathway Analysis of DEGs in BAR-T Cells.

Pepsin vs. Sham		
**Top Canonical Pathways**	***p*-value**	**Overlap**
Keratinization	2.75 × 10^−6^	4/214 (1.9%)
Wound Healing Signaling Pathway	2.27 × 10^−4^	3/252 (1.2%)
Glucocorticoid Receptor Signaling	2.57 × 10^−3^	3/582 (0.5%)
**Top Diseases**	***p*-value range**	**#Genes**
Dermatological Diseases and Conditions	2.35 × 10^−3^–4.94 × 10^−4^	2
Developmental Disorder	2.96 × 10^−3^–4.94 × 10^−4^	2
Organismal Injury and Abnormalities	4.72 × 10^−2^–4.94 × 10^−4^	12
**Molecular and Cellular Functions**	***p*-value range**	**#Genes**
Cell Morphology	4.79 × 10^−2^–1.23 × 10^−5^	3
Cellular Assembly and Organization	3.22 × 10^−2^–1.48 × 10^−4^	4
Cellular Function and Maintenance	4.65 × 10^−2^–1.48 × 10^−4^	4
**Top Upstream Regulators**	***p*-value range**	
CAMK4	2.44 × 10^−4^	
miR-7002-5p (miRNAs w/seed UGGCUUC)	4.98 × 10^−4^	
cytisine	4.98 × 10^−4^	
**Pepsin + Amprenavir vs. Sham**		
**Top Canonical Pathways**	***p*-value**	**Overlap**
Keratinization	7.15 × 10^−7^	4/214 (1.9%)
Glucocorticoid Receptor Signaling	1.03 × 10^−3^	3/582 (0.5%)
Wound Healing Signaling Pathway	3.68 × 10^−3^	2/252 (0.8%)
**Top Diseases**	***p*-value range**	**#Genes**
Cancer	3.61 × 10^−2^–3.71 × 10^−4^	8
Dermatological Diseases and Conditions	3.54 × 10^−2^–3.71 × 10^−4^	3
Gastrointestinal Disease	4.07 × 10^−2^–3.71 × 10^−4^	3
**Molecular and Cellular Functions**	***p*-value range**	**#Genes**
Cell Morphology	2.82 × 10^−2^–6.71 × 10^−6^	3
Cellular Assembly and Organization	2.78 × 10^−2^–8.09 × 10^−5^	3
Cellular Function and Maintenance	1.58 × 10^−2^–8.09 × 10^−5^	3
**Top Upstream Regulators**	***p*-value range**	
GSTP1	2.37 × 10^−5^	
CREB:CRTC1:PER1 gene	3.45 × 10^−4^	
BMAL1:CLOCK,NPAS2:PER1 gene	3.45 × 10^−4^	
**Pepsin + Amprenavir vs. Pepsin**		
**Top Canonical Pathways**	***p*-value**	**Overlap**
Wound Healing Signaling Pathway	9.44 × 10^−8^	7/252 (2.8%)
HMGB1 Signaling	5.30 × 10^−6^	5/167 (3.0%)
Macrophage Classical Activation Signaling Pathway	9.67 × 10^−6^	5/189 (2.6%)
**Top Diseases**	***p*-value range**	**#Genes**
Cancer	1.00 × 10^−2^–7.95 × 10^−8^	37
Immunological Disease	9.79 × 10^−3^–7.95 × 10^−8^	25
Organismal Injury and Abnormalities	1.00 × 10^−2^–7.95× 10^−8^	37
**Molecular and Cellular Functions**	***p*-value range**	**#Genes**
Cell-To-Cell Signaling and Interaction	9.11 × 10^−3^–1.08 × 10^−7^	19
Lipid Metabolism	9.11 × 10^−3^–3.68 × 10^−7^	15
Cellular Development	9.53 × 10^−3^–6.86 × 10^−7^	22
**Top Upstream Regulators**	***p*-value range**	
TBK1	1.40 × 10^−8^	
SB203580	2.85 × 10^−8^	
VEGF	4.44 × 10^−8^	

IPA enrichment of DEGs from pepsin vs. sham, pepsin + amprenavir vs. sham, and pepsin + amprenavir vs. pepsin, showing top pathways, diseases/functions, and upstream regulators with Fisher’s exact test *p* values and gene overlap (%).

**Table 4:** Top DEGs in BAR-T Cells.

Pepsin vs. Sham	logFC		Pepsin + Amprenavir vs. Sham	logFC		Pepsin + Amprenavir vs. Pepsin	LogFC	
**FOSB**	−0.45	↓	**PER1**	−0.46	↓	**RND1**	−0.69	↓
**AVIL**	−0.84	↓	**RND1**	−0.56	↓	**KRT84**	−5.16	↓
**KRT6B**	−1.03	↓	**KRT6B**	−1.02	↓	**CHD7**	−0.46	↓
**GOLGA8A-B**	−0.52	↓	**RASGEF1B**	−0.62	↓	**RNF152**	−0.49	↓
**ATG9B**	−0.59	↓	**KRTAP2−4**	0.87	↑	**REL**	−0.50	↓
**CPT1B**	−0.51	↓	**CEACAM5**	0.51	↑	**CLCF1**	0.50	↑
**AHSA2P**	−0.55	↓	**KRT71**	6.24	↑	**KRTAP2−4**	0.71	↑
**KRT71**	4.61	↑	**KRT72**	6.02	↑	**RGCC**	0.51	↑
**KRT72**	4.37	↑	**PRR15L**	0.54	↑	**YJEFN3**	0.83	↑
						**DOK3**	0.68	↑

List of differentially expressed genes (DEGs) with log fold changes (logFC) in BAR-T cells under different conditions: pepsin vs. sham, pepsin + amprenavir vs. sham, and pepsin + amprenavir vs. pepsin. Genes with upregulation are marked with ↑, and downregulation with ↓. The table shows the logFC for each DEG under the respective conditions.

## Data Availability

The raw RNA-seq data presented in this study are publicly available in the Gene Expression Omnibus (GEO) database under the accession number GSE288541.

## Supplementary Materials

The following supporting information can be downloaded at https://www.mdpi.com/article/10.3390/ijms26136182/s1.

## Appendix A

*ADM2* (Adrenomedullin 2), *AHSA2P* (AHSA2 pseudogene), *ATG9B* (Autophagy Related 9B), *AVIL* (Avilin), *BMAL1:CLOCK, NPAS2:PER1* gene (BMAL1, Clock Circadian Regulator, NPAS2, Period Circadian Regulator 1), *CAMK4* (Calcium/Calmodulin Dependent Protein Kinase IV), *CAV1* (Caveolin 1), *CEACAM5* (Carcinoembryonic Antigen Related Cell Adhesion Molecule 5), *CHAC1* (ChaC Glutathione S-Transferase 1), *CHD7* (Chromodomain Helicase DNA Binding Protein 7), *CLCF1* (Cytokine-Like Factor 1), *COL1A2* (Collagen Type I Alpha 2 Chain), *CPT1B* (Carnitine Palmitoyltransferase 1B), *CREB:CRTC1:PER1* gene (CREB Binding Protein, CREB Regulated Transcription Coactivator 1, Period Circadian Regulator 1), *CXCL14* (C-X-C Motif Chemokine Ligand 14), *DAP3* (Death Associated Protein 3), *DOK3* (Docking Protein 3), *EIF2S1* (Eukaryotic Translation Initiation Factor 2 Subunit 1), *FOS* (Fos Proto-Oncogene), *FOSB* (Fos Proto-Oncogene B), *GLI1* (GLI Family Zinc Finger 1), *GOLGA8A-B* (Golgin Subfamily A Member 8A-B), *GSTP1* (Glutathione S-Transferase Pi 1), *HES1* (Hairy And Enhancer Of Split 1), *HGF* (Hepatocyte Growth Factor), *HSPA1A/HSPA1B* (Heat Shock Protein Family A (Hsp70) Member 1A/1B), *ID1* (Inhibitor Of DNA Binding 1), *IL11* (Interleukin 11), *KRT6B* (Keratin 6B), *KRT71* (Keratin 71), *KRT72* (Keratin 72), *KRT84* (Keratin 84), *KRTAP2-4* (Keratin Associated Protein 2-4), *MIDN* (Midline 1), *MT-CO1* (Mitochondrial Cytochrome C Oxidase Subunit 1), *MT-CO2* (Mitochondrial Cytochrome C Oxidase Subunit 2), *MT-CYB* (Mitochondrial Cytochrome B), *MT-ND1* (Mitochondrial NADH Dehydrogenase 1), *MT-ND2* (Mitochondrial NADH Dehydrogenase 2), *MT-ND4* (Mitochondrial NADH Dehydrogenase 4), *mtRNase P* (Mitochondrial RNase P), *NSUN3* (Nucleolar RNA Methyltransferase 3), *PER1* (Period Circadian Regulator 1), *POTEE/POTEF* (Potassium Channel Tetramerization Domain Containing 1/Potassium Channel Tetramerization Domain Containing 2), *PRL* (Prolactin), *PRR15L* (Proline Rich 15-Like), *RASGEF1B* (RAS Guanyl Releasing Protein 1B), *REL* (RELA Proto-Oncogene), *RGCC* (Regulator of Cell Cycle), *RND1* (Rho Family GTPase 1), *RNF152* (Ring Finger Protein 152), *SAA2* (Serum Amyloid A2), *SAA2-SAA4* (Serum Amyloid A2/4), *SB203580* (P38 MAPK Inhibitor), *SESN2* (Sestrin 2), *SGPP2* (Sphingosine-1-Phosphate Phosphatase 2), *SLC7A11* (Solute Carrier Family 7 Member 11), *STC2* (Stanniocalcin 2), *TBK1* (TANK-Binding Kinase 1), *VEGF* (Vascular Endothelial Growth Factor), *YJEFN3* (YJF3 Protein).
