# Supplementary figures and images for: Amprenavir Mitigates Pepsin-Induced Transcriptomic Changes in Normal and Precancerous Esophageal Cells
Source: Int J Mol Sci. 2025 Jun 26;26(13):6182. doi: 10.3390/ijms26136182 (PMC12250232; doi:10.3390/ijms26136182)

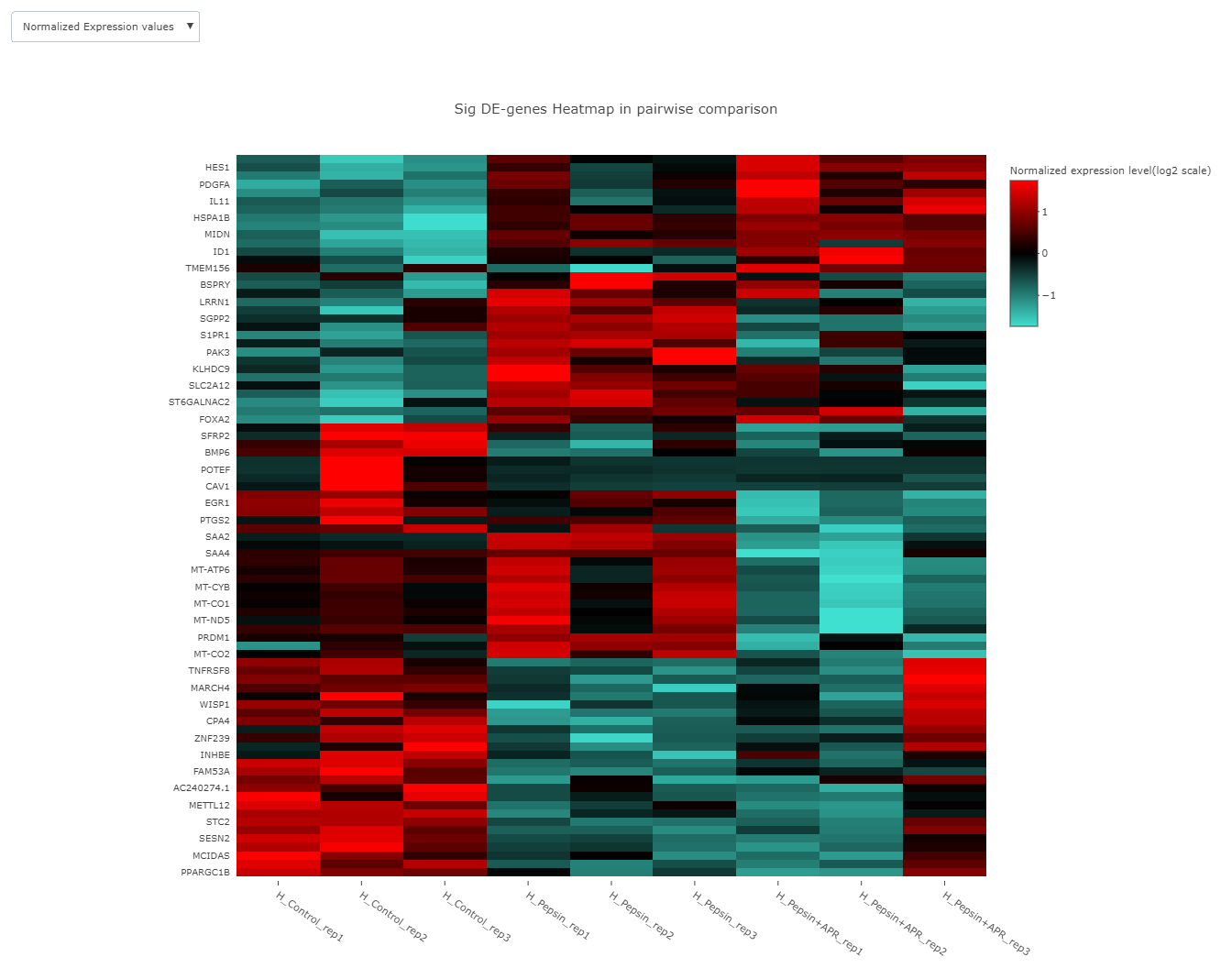

Supplement: Supplementary file 1 [file ijms-26-06182-s001.zip › Suppplementary Figure S3_heatmap_Het-1A.png]

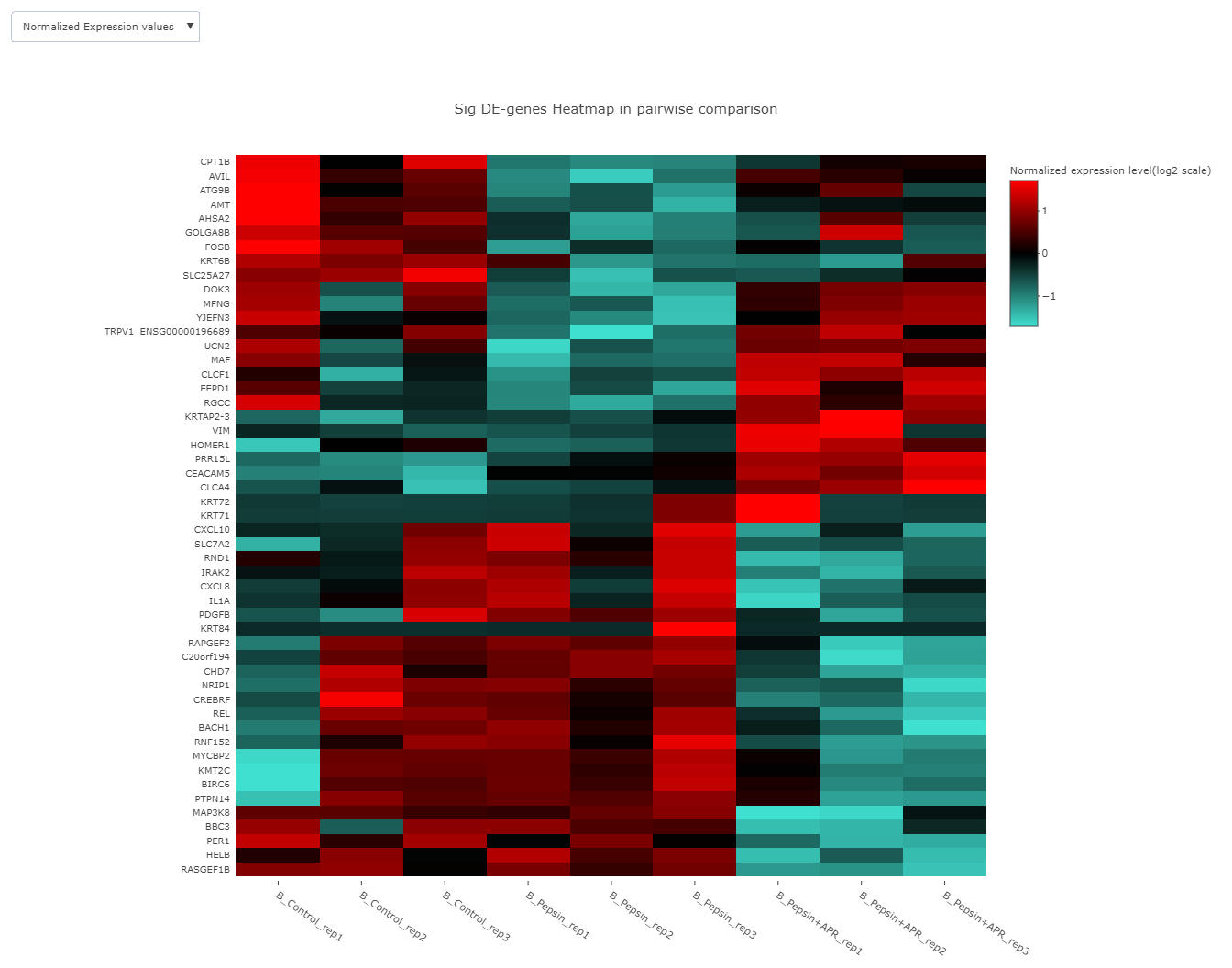

Supplement: Supplementary file 1 [file ijms-26-06182-s001.zip › Suppplementary Figure S4_heatmap_BAR-T.png]

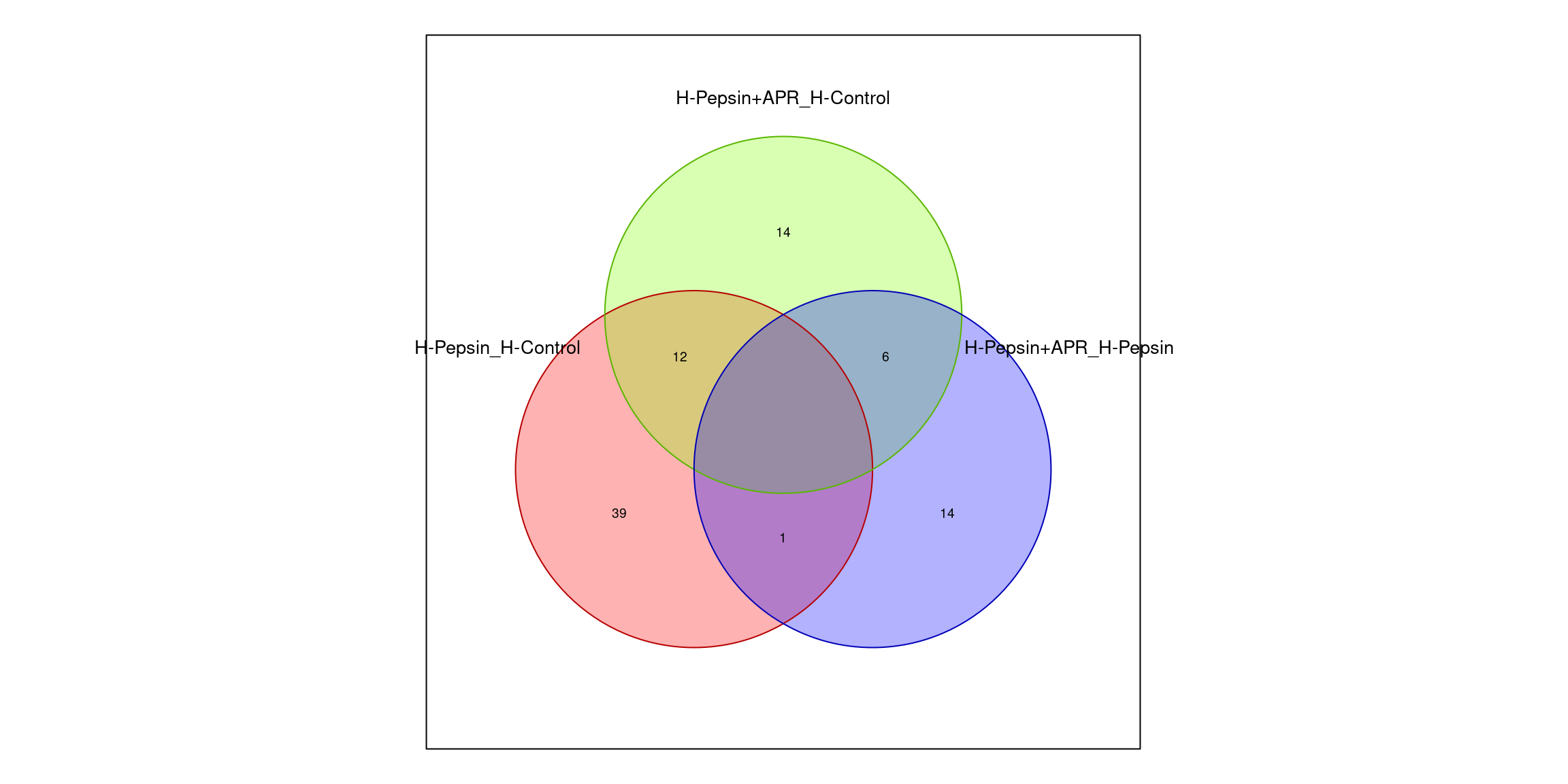

Supplement: Supplementary file 1 [file ijms-26-06182-s001.zip › Suppplementary Figure S1_vendiagramclassic -Het-1A.png]

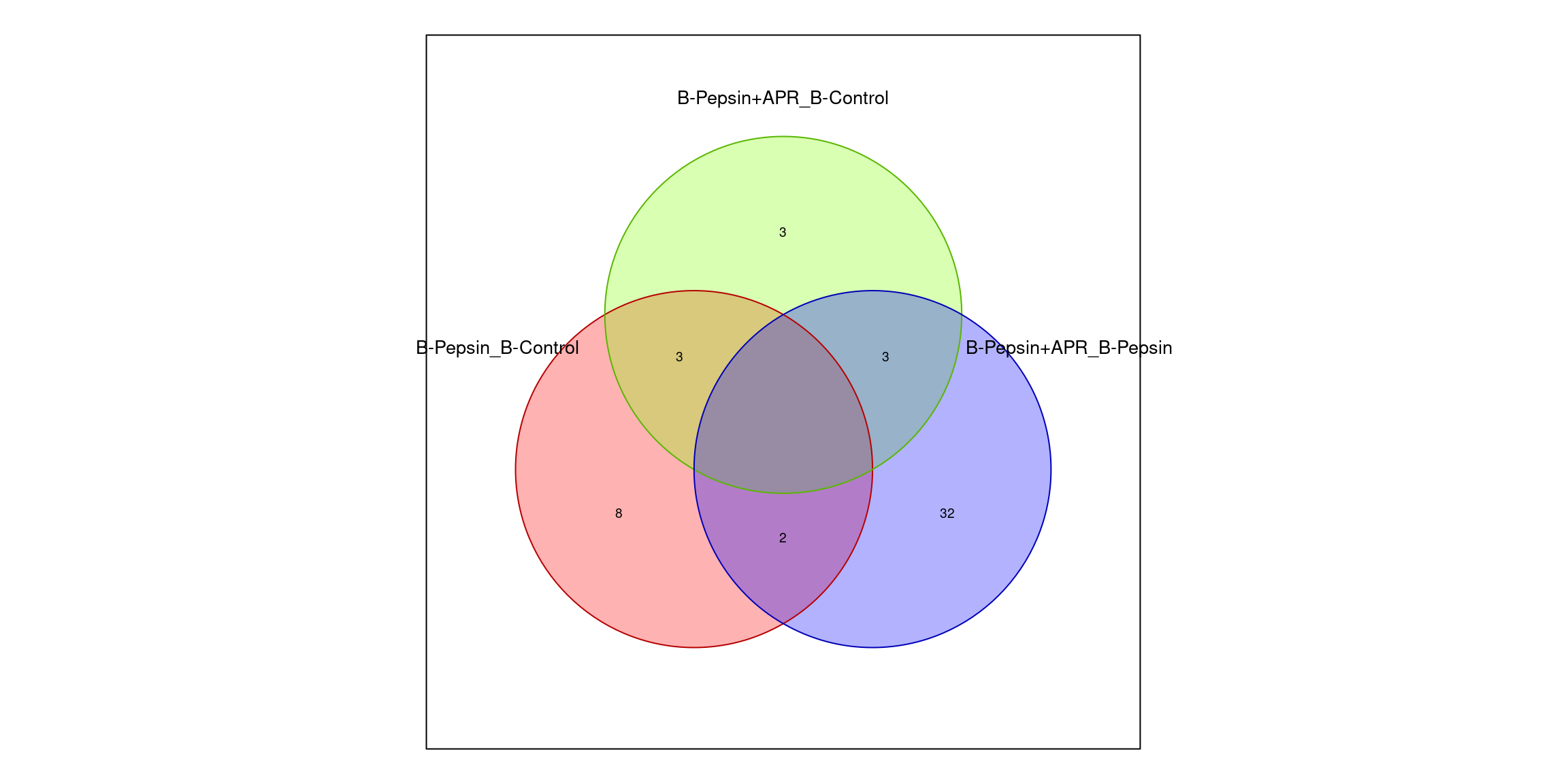

Supplement: Supplementary file 1 [file ijms-26-06182-s001.zip › Suppplementary Figure S2_vendiagramclassic -1_BAR-T.png]
